# Supplementary material for: Analysis of PPARγ Signaling Activity in Psoriasis
Source: Int J Mol Sci. 2021 Aug 10;22(16):8603. doi: 10.3390/ijms22168603 (PMC8395241; doi:10.3390/ijms22168603)
Supplement: Supplementary file 1 [file ijms-22-08603-s001.zip › Supplemental materials_Analysis of PPARg signaling activity in psoriasis/Pathway models/Models images and html files/Model of PPARG related pathways in psoriasis (short version)/4456.html]

FOXO1


# Protein FOXO1

|  |  |
| --- | --- |
| URN | urn:agi-llid:2308 |
| Total Entities | 0 |
| Connectivity | 4482 |
| Name | FOXO1 |
| Description | forkhead box O1 |
| Notes | This gene belongs to the forkhead family of transcription factors which are characterized by a distinct forkhead domain. The specific function of this gene has not yet been determined; however, it may play a role in myogenic growth and differentiation. Translocation of this gene with PAX3 has been associated with alveolar rhabdomyosarcoma. [provided by RefSeq, Jul 2008] |
| Primary Cell Localization | Nucleus |
| Class | Transcription factor |
| ObjectType | Protein |

---

|  |  |
| --- | --- |
| Pathway | Androgen Deficiency in Male Obesity |
|  | SIRT2 Signaling in Aging |
|  | SIRT7 Signaling in Aging |
|  | SIRT1 Signaling in Aging |
|  | MTOR/TP53 Regulates Cell Metabolism |
|  | Insulin Influence on Protein Synthesis |
|  | Insulin Signaling |
|  | Androgens in Adipocyte Activation |
|  | LH and FSH Signaling and Steroidogenesis |
|  | V(D)J Recombination Activation |
|  | Adipokines Production by Adipocyte |
|  | Lipolysis Regulation in Adipocyte |
|  | SCF/SKP2 Complex |
|  | Proteins Involved in Hypertrophic Cardiomyopathy |
|  | Proteins Involved in Acne Vulgaris |
|  | Hyperseborrhea in Acne Vulgaris |
|  | Androgen Induced Sebocyte Hyperfunction |
|  | Androgens in Sebocyte Maturation |
|  | Proteins Involved in Diabetes Mellitus Type 2 |
|  | Donohue Syndrome Progression (Hypothesis) |
|  | Proteins Involved in Insulin Resistance |
|  | Adiponectin Synthesis Declines in Insulin Resistance |
|  | Proteins Involved in non-Alcoholic Fatty Liver Disease |
|  | Gluconeogenesis Impairment in non-Alcoholic Fatty Liver Disease |
|  | PNPLA3 in non-Alcoholic Fatty Liver Disease |
|  | FOXO1 and SREBP-1C Role in beta-Cell Suppression (Rodent Model) |
|  | Myocyte Dysfunction in Hypoparathyroidism |
|  | Adipocyte Hypertrophy and Hyperplasia |
|  | Adipokines Production by Adipocyte Impaired in Obesity |
|  | Peutz-Jeghers Syndrome |
|  | B-Cell Acute Lymphoblastic Leukemia |
|  | B-Cell Chronic Lymphocytic Leukemia |
|  | T-Cell Acute Lymphoblastic Leukemia |
|  | Proteins Involved in Hepatocellular Carcinoma |
|  | Hepatocellular Carcinoma |
|  | Growth Factor Signaling in Hepatocellular Carcinoma |
|  | Pancreatic Ductal Carcinoma |
|  | Growth Factor Signaling in Pancreatic Neoplasms |
|  | Ovulation Block |
|  | SHBG and IGFBP1 Synthesis Decline in Polycystic Ovary Syndrome |
|  | Polycystic Ovary Syndrome |
|  | FSH Action in Polycystic Ovary Syndrome |
|  | TNF Receptor Associated Periodic Syndrome |
|  | Cowden Syndrome |
|  | ER Stress in Huntington Disease |
|  | Ca2+ Overload in Muscular Dystrophies |
|  | Muscular Dystrophies |
|  | Dystrophin Glycoprotein Complex Signaling in Muscular Dystrophies |
|  | IGF1 Role in Muscle Hypertrophy |
|  | Myostatin-IGF1 Crosstalk in Skeletal Muscles in Muscular Dystrophies |
|  | Proteins Involved in Medulloblastoma |
|  | ERBB/VEGFR/Akt Signaling in Breast Cancer |
|  | IGF1R/AKT Signaling in Breast Cancer |
|  | ESR1/ERBB Positive Luminal Breast Cancer |
|  | Breast Cancer |
|  | ESR1 Signaling in Breast Cancer |
|  | Endometrial Cancer |
|  | Endometrioid Endometrial Cancer |
|  | Clear Cell Endometrial Cancer and Papillary Serous Endometrial Cancer |
|  | Proteins with Altered Expression in Endometriosis |
|  | Progesterone Resistance in Endometriosis |
|  | Endometrioid Ovarian Carcinoma |
|  | Clear Cell Ovarian Carcinoma |
|  | High-Grade Serous Ovarian Carcinoma |
|  | Low-Grade Serous Ovarian Carcinoma |
|  | Ovarian Cancer |
|  | Mucinous Ovarian Carcinoma |
|  | Proteins Involved in Primary Ovarian Insufficiency |
|  | LH and FSH Signaling Impairment in Primary Ovarian Insufficiency |
|  | Androgen Receptor/Akt Signaling |
|  | Alveolar Epithelial Cell Autophagy in COPD |
|  | FSHR Expression Targets |
|  | PI3K/AKT/MTOR Signaling Activation in Cancer |
|  | PI3K/AKT/MTOR Signaling Activation by Blocking of Tumor Suppressors |
|  | ANGPT/TEK Maintains Endothelial Cell Quiescence in Cancer |
|  | ANGPT/TEK Stimulates Endothelial Cell Migration in Cancer |
|  | Skeletal Muscle Wasting in Cancer Cachexia |
|  | IGF1 -> CEBPA/FOXO1A Expression Targets |
|  | IGF2 -> CEBPA/FOXO1A Expression Targets |
|  | Insulin -> STAT Expression Targets |
|  | Insulin -> CEBPA/CTNNB/FOXA/FOXO Expression Targets |
|  | ICAM2 -> CTNNB/FOXO/STAT3 Signaling |
|  | InsulinR -> CTNNB/FOXA/FOXO Signaling |
|  | IGF1R -> CEBPA/FOXO1A Signaling |
|  | IL7R -> FOXO/NF-kB Signaling |
|  | IL7 Expression Targets |
|  | Leptin -> STAT Expression Targets |
|  | NGF -> FOXO/MYCN/ELK-SRF Expression Targets |
|  | BDNF Expression Targets |
|  | NTF3 Expression Targets |
|  | AngiopoietinR -> FOXO Signaling |
|  | NTRK -> FOXO/MYCN Signaling |
|  | Androgen Receptor non-Genomic Signaling |
|  | FOXO1 Signaling |
|  | AMPK Signaling |
|  | prarg negative regulators, ps-positive |
|  | PPARG negative regulators |
|  | pparg expr target\_regulators |
|  | Neighbors of disease exacerbation |
|  | Neighbors of tumor progression |
|  | Neighbors of cancer progression |
|  | Neighbors of overall survival |
|  | Neighbors of disease severity |
|  | Neighbors of lesion size |
|  | Neighbors of mortality |
|  | Neighbors of survival rate |
|  | Neighbors of infarct size |
|  | Neighbors of survival time |
|  | Neighbors of insulin sensitivity |
|  | Neighbors of graft survival |
|  | Neighbors of lethality |
|  | Neighbors of recurrence-free survival |
|  | Neighbors of long-term survival |
|  | Neighbors of bone mass |
|  | Neighbors of body weight |
|  | Neighbors of body weight gain |
|  | Neighbors of glucose tolerance |
|  | Neighbors of fat mass |
|  | Neighbors of exhaustion |
|  | Neighbors of bone mineral density |
|  | Neighbors of shear stress |
|  | Neighbors of viral load |
|  | Neighbors of blood pressure |
|  | Neighbors of muscle mass |
|  | Neighbors of tumor aggressiveness |
|  | Neighbors of systolic blood pressure |
|  | Neighbors of blood glucose |
|  | Neighbors of renal hypertrophy |
|  | Neighbors of fasting plasma glucose |
|  | Neighbors of osteoclast number |
|  | Neighbors of T-cell count |
|  | Neighbors of energy expenditure |
|  | Neighbors of glucose level |
|  | Neighbors of left ventricular ejection fraction |
|  | Neighbors of proliferation index |
|  | Neighbors of fertility |
|  | Neighbors of metabolic response |
|  | Neighbors of insulin level |
|  | Neighbors of carotid intima-media thickness |
|  | Neighbors of bone volume |
|  | Neighbors of fractional shortening |
|  | Neighbors of disease exacerbation |
|  | Neighbors of tumor progression |
|  | Neighbors of cancer progression |
|  | Neighbors of overall survival |
|  | Neighbors of disease severity |
|  | Neighbors of lesion size |
|  | Neighbors of mortality |
|  | Neighbors of survival rate |
|  | Neighbors of infarct size |
|  | Neighbors of survival time |
|  | Neighbors of insulin sensitivity |
|  | Neighbors of graft survival |
|  | Neighbors of lethality |
|  | Neighbors of recurrence-free survival |
|  | Neighbors of long-term survival |
|  | Neighbors of bone mass |
|  | Neighbors of body weight |
|  | Neighbors of body weight gain |
|  | Neighbors of glucose tolerance |
|  | Neighbors of fat mass |
|  | Neighbors of exhaustion |
|  | Neighbors of bone mineral density |
|  | Neighbors of shear stress |
|  | Neighbors of viral load |
|  | Neighbors of blood pressure |
|  | Neighbors of muscle mass |
|  | Neighbors of tumor aggressiveness |
|  | Neighbors of systolic blood pressure |
|  | Neighbors of blood glucose |
|  | Neighbors of renal hypertrophy |
|  | Neighbors of fasting plasma glucose |
|  | Neighbors of osteoclast number |
|  | Neighbors of T-cell count |
|  | Neighbors of energy expenditure |
|  | Neighbors of glucose level |
|  | Neighbors of left ventricular ejection fraction |
|  | Neighbors of proliferation index |
|  | Neighbors of fertility |
|  | Neighbors of metabolic response |
|  | Neighbors of insulin level |
|  | Neighbors of carotid intima-media thickness |
|  | Neighbors of bone volume |
|  | Neighbors of fractional shortening |
|  | Neighbors of cell development |
|  | Neighbors of immune response |
|  | Neighbors of cell population |
|  | Neighbors of T-cell development |
|  | Neighbors of inflammatory response |
|  | Neighbors of wound healing |
|  | Neighbors of angiogenesis |
|  | Neighbors of innate immune response |
|  | Neighbors of cell function |
|  | Neighbors of macrophage activation |
|  | Neighbors of cell formation |
|  | Neighbors of adaptive immune response |
|  | Neighbors of osteoclast development |
|  | Neighbors of epithelial to mesenchymal transition |
|  | Neighbors of immunity |
|  | Neighbors of cell proliferative response |
|  | Neighbors of chemotaxis |
|  | Neighbors of cell survival |
|  | Neighbors of osteoclast differentiation |
|  | Neighbors of cell phenotype |
|  | Neighbors of T-cell proliferation |
|  | Neighbors of cell adhesion |
|  | Neighbors of T-cell activation |
|  | Neighbors of endothelial cell proliferation |
|  | Neighbors of fibrogenesis |
|  | Neighbors of tumor growth |
|  | Neighbors of tissue repair |
|  | Neighbors of cell count |
|  | Neighbors of cell interaction |
|  | Neighbors of SMC proliferation |
|  | Neighbors of lumen formation |
|  | Neighbors of endothelial cell migration |
|  | Neighbors of adipogenesis |
|  | Neighbors of lipid storage |
|  | Neighbors of cell damage |
|  | Neighbors of pregnancy |
|  | Neighbors of aging |
|  | Neighbors of cell motility |
|  | Neighbors of fibroblast proliferation |
|  | Neighbors of adipocyte differentiation |
|  | Neighbors of microglial activation |
|  | Neighbors of regeneration |
|  | Neighbors of cell migration |
|  | Neighbors of cancer growth |
|  | Neighbors of stem cell proliferation |
|  | Neighbors of hepatic regeneration |
|  | Neighbors of phagocytosis |
|  | Neighbors of immune system activation |
|  | Neighbors of leukocyte migration |
|  | Neighbors of osteoclast formation |
|  | Neighbors of cell invasion |
|  | Neighbors of heart function |
|  | Neighbors of bone remodeling |
|  | Neighbors of ossification |
|  | Neighbors of T-cell function |
|  | Neighbors of immune system function |
|  | Neighbors of autophagy |
|  | Neighbors of monocyte adhesion |
|  | Neighbors of transcription activation |
|  | Neighbors of senescence |
|  | Neighbors of T-cell response |
|  | Neighbors of hepatocyte proliferation |
|  | Neighbors of cell transdifferentiation |
|  | Neighbors of neuroprotection |
|  | Neighbors of stem cell differentiation |
|  | Neighbors of endothelial cell function |
|  | Neighbors of neuronal death |
|  | Neighbors of cell growth |
|  | Neighbors of ROS generation |
|  | Neighbors of cancer cell growth |
|  | Neighbors of sensitization |
|  | Neighbors of hemato-encephalic barrier |
|  | Neighbors of macrophage function |
|  | Neighbors of cellular immune response |
|  | Neighbors of cellular senescence |
|  | Neighbors of colony formation |
|  | Neighbors of cell differentiation |
|  | Neighbors of keratinocyte proliferation |
|  | Neighbors of life span |
|  | Neighbors of cell homeostasis |
|  | Neighbors of psoriasis |
|  | Neighbors of inflammatory disease |
|  | Neighbors of rheumatoid arthritis |
|  | Neighbors of atherosclerosis |
|  | Neighbors of fibrosis |
|  | Neighbors of inflammatory bowel disease |
|  | Neighbors of injury |
|  | Neighbors of vascular remodeling |
|  | Neighbors of metastasis |
|  | Neighbors of carcinogenesis |
|  | Neighbors of inflammation |
|  | Neighbors of tumor microenvironment |
|  | Neighbors of obesity |
|  | Neighbors of vascular disease |
|  | Neighbors of diabetes mellitus |
|  | Neighbors of asthma |
|  | Neighbors of pneumonia |
|  | Neighbors of hyperplasia |
|  | Neighbors of liver fibrosis |
|  | Neighbors of chronic obstructive pulmonary disease |
|  | Neighbors of myocardial infarction |
|  | Neighbors of reperfusion injury |
|  | Neighbors of hepatitis |
|  | Neighbors of insulin resistance |
|  | Neighbors of infection |
|  | Neighbors of vascular injury |
|  | Neighbors of renal injury |
|  | Neighbors of liver injury |
|  | Neighbors of neuroinflammation |
|  | Neighbors of sepsis |
|  | Neighbors of death |
|  | Neighbors of type 1 diabetes |
|  | Neighbors of encephalomyelitis |
|  | Neighbors of autoimmune disease |
|  | Neighbors of ischemic stroke |
|  | Neighbors of neutrophil infiltration |
|  | Neighbors of heart disease |
|  | Neighbors of diabetic nephropathy |
|  | Neighbors of lung injury |
|  | Neighbors of hepatocellular carcinoma |
|  | Neighbors of autoimmunity |
|  | Neighbors of gastric cancer |
|  | Neighbors of colorectal cancer |
|  | Neighbors of cardiac remodeling |
|  | Neighbors of breast cancer |
|  | Neighbors of ischemia |
|  | Neighbors of neoplasm invasion |
|  | Neighbors of kidney disease |
|  | Neighbors of heart failure |
|  | Neighbors of cardiovascular disease |
|  | Neighbors of stroke |
|  | Neighbors of cancer |
|  | Neighbors of cerebral ischemia |
|  | Neighbors of type 2 diabetes |
|  | Neighbors of neoplasm |
|  | Neighbors of atrophy |
|  | Neighbors of diet-induced obesity |
|  | Neighbors of myocardial fibrosis |
|  | Neighbors of endothelial cell dysfunction |
|  | Neighbors of liver disease |
|  | Neighbors of prostate cancer |
|  | Neighbors of hypertrophy |
|  | Neighbors of osteolysis |
|  | Neighbors of ovarian cancer |
|  | Neighbors of proteinuria |
|  | Neighbors of monocyte |
|  | Neighbors of macrophage |
|  | Neighbors of hepatocyte |
|  | Neighbors of endothelial cell |
|  | Neighbors of peritoneal macrophage |
|  | Neighbors of stem cell |
|  | Neighbors of insulin-secreting cell |
|  | Neighbors of T-cell |
|  | Neighbors of liver |
|  | Neighbors of skin |
|  | Neighbors of intestine |
|  | Neighbors of blood vessel |
|  | Neighbors of heart |
|  | Neighbors of microvessel |
|  | Neighbors of uterus |
|  | Neighbors of esophagus |
|  | Neighbors of central nervous system |
|  | Neighbors of lymphatic system |
|  | Neighbors of NF-kB family |
|  | Neighbors of oxidized LDL |
|  | Neighbors of JNK |
|  | Neighbors of PPAR |
|  | Neighbors of Jun/Fos |
|  | Neighbors of PI3K |
|  | Neighbors of STAT family |
|  | Neighbors of PDGF |
|  | Neighbors of ERK1/2 |
|  | Neighbors of growth factor |
|  | Neighbors of chorionic gonadotropin |
|  | Neighbors of IKBK |
|  | Neighbors of AMPK |
|  | Neighbors of T-cell receptor |
|  | Neighbors of histone deacetylase |
|  | Neighbors of HIF-1 |
|  | Neighbors of IL12 |
|  | Neighbors of TSH |
|  | Neighbors of proteasome endopeptidase complex |
|  | Neighbors of non-coding RNA |
|  | Neighbors of STAT5 |
|  | Neighbors of gonadotropins |
|  | Neighbors of FSH |
|  | Neighbors of histone |
|  | Neighbors of histone acetyltransferase |
|  | Neighbors of deacetylase |
|  | Model of PPARG signaling in psoriasis |
|  | PPARG negative regulators and targets |
|  | Model of PPARG related pathways in psoriasis (short version) |
|  | H2A-K119 and H3-K27 |
|  | New Pathway |
|  | 3\_Increased proliferation of sebocytes in acne vulgaris |
|  | 2\_Progesterone resistance stimulates endometriotic cell survival |
|  | 2\_Impaired steroidogenesis in PCOS |
|  | 2\_Cigarette smoke and inflammation cause alveolar epithelial cell death in COPD |
|  | 1\_1\_Insulin synthesis and secretion: insulin processing and storage |
|  | 3\_3\_Beta-cell proliferation and compensation: recovery |
|  | 4\_1\_Insulin resistance: insulin signaling |
|  | 4\_3\_Insulin resistance: FFA-related insulin resistance |

---

|  |  |
| --- | --- |
| Group | Genes with Mutations Associated with Ageing (non-Mammalian Animal Model) |
|  | Proteins Involved in Cowden Syndrome |

---

|  |  |
| --- | --- |
| MedScan ID | 2308 |

---

|  |  |
| --- | --- |
| LocusLink ID | 2308 |
|  | 56458 |
|  | 84482 |

---

|  |  |
| --- | --- |
| Alias | forkhead homologue in rhabdomyosarcoma |
|  | FOXO1A |
|  | forkhead homolog in rhabdomyosarcoma |
|  | forkhead box O1A |
|  | forkhead homolog I |
|  | Forkhead in rhabdomyosarcoma |
|  | transcription factor FKHR |
|  | FKH I |
|  | transcription factor FOXO 1A |
|  | forkhead protein FKHR |
|  | forkhead transcription factor FKHR |
|  | AI876417 |
|  | forkhead, Drosophila, homolog of, in rhabdomyosarcoma |
|  | transcription factor FOXO 1 |
|  | FKHR |
|  | Fkhr1 |
|  | forkhead (Drosophila) homolog 1 (rhabdomyosarcoma) |
|  | homolog of Drosophila forkhead |
|  | forkhead homolog 1 (rhabdomyosarcoma) |
|  | OTTHUMP00000018301 |
|  | forkhead protein I |
|  | Forkhead related transcription factor |
|  | Foxo I |
|  | forkhead box O1 |
|  | forkhead protein 1 |
|  | forkhead box O1A (rhabdomyosarcoma) |
|  | OTTMUSP00000023486 |
|  | FKH1 |
|  | forkhead homolog 1 |
|  | Fkhr I |
|  | Foxo1 |
|  | FOX O1A |
|  | forkhead box protein O1A |
|  | forkhead box protein O1 |
|  | Afxh |

---

|  |  |
| --- | --- |
| GO ID | 0003700 |
|  | 0000981 |
|  | 0001227 |
|  | 0008013 |
|  | 0003682 |
|  | 0051721 |
|  | 0043565 |
|  | 0001223 |
|  | 0008134 |
|  | 0031625 |
|  | 0006915 |
|  | 0006914 |
|  | 0001568 |
|  | 0001678 |
|  | 0006974 |
|  | 0070417 |
|  | 0071549 |
|  | 0070301 |
|  | 0071455 |
|  | 0032869 |
|  | 0071732 |
|  | 0034599 |
|  | 0009267 |
|  | 0019221 |
|  | 0070166 |
|  | 0031018 |
|  | 0097009 |
|  | 0045444 |
|  | 0042593 |
|  | 0008286 |
|  | 0043066 |
|  | 0090090 |
|  | 1903243 |
|  | 0045599 |
|  | 0032873 |
|  | 0000122 |
|  | 0045892 |
|  | 0097150 |
|  | 0043065 |
|  | 0010508 |
|  | 0045722 |
|  | 0045732 |
|  | 0045944 |
|  | 0045893 |
|  | 0006473 |
|  | 0035947 |
|  | 2000177 |
|  | 2000377 |
|  | 1902617 |
|  | 0001659 |
|  | 0005737 |
|  | 0005829 |
|  | 0005739 |
|  | 0005654 |
|  | 0005634 |
|  | 0003677 |
|  | 0030154 |
|  | 0042127 |
|  | 0006357 |
|  | 0006355 |
|  | 0010629 |
|  | 0018105 |
|  | 0018107 |
|  | 1903661 |
|  | 0032868 |
|  | 0005667 |
|  | 0045087 |
|  | 0006916 |
|  | 0006351 |
|  | 0048513 |
|  | 0005515 |
|  | 0019901 |
|  | 0000989 |
|  | 0007173 |
|  | 0009790 |
|  | 0003690 |
|  | 0038095 |
|  | 0006366 |
|  | 0008543 |
|  | 0051090 |
|  | 0008301 |
|  | 0001078 |
|  | 0048011 |
|  | 0003705 |
|  | 2001141 |
|  | 0048015 |
|  | 0007389 |
|  | 0009888 |
|  | 1903506 |
|  | 2000505 |
|  | 0044429 |
|  | 0010843 |
|  | 0003704 |
|  | 0016566 |
|  | 0016563 |
|  | 0010553 |
|  | 0006350 |
|  | 0045941 |
|  | 0005575 |

---

|  |  |
| --- | --- |
| KEGG ID | hsa:2308 |
|  | mmu:56458 |
|  | rno:84482 |

---

|  |  |
| --- | --- |
| Organism | Homo sapiens {Organism urn:agi-taxid:9606} |
|  | Mus musculus {Organism urn:agi-taxid:10090} |
|  | Rattus norvegicus {Organism urn:agi-taxid:10116} |
|  | Homo sapiens |
|  | Mus musculus |
|  | Rattus norvegicus |

---

|  |  |
| --- | --- |
| Mouse chromosome position | 3 23.19 cM |
|  | 3 22.5 cM |

---

|  |  |
| --- | --- |
| OMIM ID | 136533 |
|  | 268220 |

---

|  |  |
| --- | --- |
| Rat chromosome position | 2q26 |

---

|  |  |
| --- | --- |
| Hugo ID | 3819 |
|  | HGNC:3819 |

---

|  |  |
| --- | --- |
| Human chromosome position | 13q14.11 |
|  | 13q14.1 |

---

|  |  |
| --- | --- |
| Swiss-Prot Accession | Q12778 |
|  | Q12778.2 |
|  | Q9R1E0 |
|  | Q9R1E0.2 |
|  | G3V7R4 |
|  | G3V7R4.1 |
|  | O43523 |
|  | Q5VYC7 |
|  | Q6NSK6 |
|  | Q9JJW4 |
|  | Q8BPP2 |
|  | Q8BRS7 |
|  | Q8CA93 |
|  | Q9WVH5 |
|  | Q9ES26 |

---

|  |  |
| --- | --- |
| PIR ID | S40521 |

---

|  |  |
| --- | --- |
| GenBank ID | NC\_000013 |
|  | NM\_002015 |
|  | NP\_002006 |
|  | XM\_011535008 |
|  | XP\_011533310 |
|  | XM\_011535010 |
|  | XP\_011533312 |
|  | NG\_023244 |
|  | AL133318 |
|  | AL355132 |
|  | AL592167 |
|  | CH471075 |
|  | EAX08627 |
|  | AF032885 |
|  | AAC39591 |
|  | AK310511 |
|  | BC021981 |
|  | AAH21981 |
|  | BC070065 |
|  | AAH70065 |
|  | BT007455 |
|  | AAP36123 |
|  | BX648278 |
|  | U02310 |
|  | AAA03629 |
|  | U36922 |
|  | Q12778 |
|  | NC\_000069 |
|  | NM\_019739 |
|  | NP\_062713 |
|  | AC104932 |
|  | AC163092 |
|  | CH466530 |
|  | EDL35224 |
|  | AF114258 |
|  | AAD42106 |
|  | AF126056 |
|  | AAD40636 |
|  | AJ252157 |
|  | CAB86873 |
|  | AK029013 |
|  | AK030304 |
|  | AK039265 |
|  | BAC30298 |
|  | AK043583 |
|  | BAC31588 |
|  | AK053633 |
|  | BAC35455 |
|  | AK054289 |
|  | AK137629 |
|  | BAE23437 |
|  | AK154041 |
|  | BAE32333 |
|  | AK185432 |
|  | AK192846 |
|  | AK211466 |
|  | AK211487 |
|  | AK212278 |
|  | AK212379 |
|  | Q9R1E0 |
|  | NC\_005101 |
|  | NM\_001191846 |
|  | NP\_001178775 |
|  | AC\_000070 |
|  | AABR07010584 |
|  | AABR07010585 |
|  | AAHX01016972 |
|  | AAHX01016973 |
|  | AAHX01016974 |
|  | CH474003 |
|  | EDM14970 |
|  | AF247812 |
|  | AAG09779 |
|  | G3V7R4 |
|  | CAI16970 |
|  | ABBA01021295 |
|  | ABBA01021296 |
|  | AABR06016643 |
|  | AABR06016644 |
|  | ABBA01021294 |
|  | AABR06016645 |
|  | U37335 |
|  | XR\_941536 |
|  | U37334 |
|  | AC\_000025 |
|  | XM\_011535009 |
|  | AAI52909 |
|  | AAHY01026720 |
|  | AAHY01026724 |
|  | AAHY01026723 |
|  | AC\_000145 |
|  | AAHY01026722 |
|  | AAHY01026721 |
|  | NC\_018924 |
|  | CAH70978 |
|  | AAHY01026719 |
|  | AMYH02027907 |
|  | XP\_011533311 |
|  | BC152908 |
|  | NT\_024524 |
|  | NW\_925473 |
|  | NW\_001838073 |
|  | AC\_000056 |
|  | NT\_039240 |
|  | NW\_001030719 |
|  | Q8BPP2 |
|  | Q8BRS7 |
|  | Q8CA93 |
|  | Q9JJW4 |
|  | Q9WVH5 |
|  | XM\_342244 |
|  | XP\_342245 |
|  | NW\_047625 |
|  | NW\_001084804 |
|  | XM\_001056726 |
|  | XP\_001056726 |
|  | Q9ES26 |

---

|  |  |
| --- | --- |
| Swiss-Prot ID | FOXO1\_HUMAN |
|  | FOXO1\_MOUSE |
|  | FOXO1\_RAT |
|  | Q9JJW4\_MOUSE |

---

|  |  |
| --- | --- |
| Cell Localization | Cytoplasm |
|  | Nucleus |

---

|  |  |
| --- | --- |
| Ensembl ID | ENSG00000150907 |
|  | ENSP00000368880.4 |
|  | ENST00000379561.6 |
|  | ENSMUSG00000044167 |
|  | ENSMUSP00000055308.5 |
|  | ENSMUST00000053764.6 |
|  | ENSRNOG00000013397 |
|  | ENSRNOP00000018244.3 |
|  | ENSRNOT00000018244.5 |
|  | ENST00000379561 |
|  | ENSRNOP00000018244 |
|  | ENSMUSP00000055308 |
|  | ENSRNOT00000018244 |
|  | ENSP00000368880 |
|  | ENST00000379561.5 |
|  | ENSMUST00000053764 |

---

|  |  |
| --- | --- |
| MGI ID | MGI:1890077 |
|  | 1890077 |

---

|  |  |
| --- | --- |
| RGD ID | 620283 |

---

|  |  |
| --- | --- |
| Unigene ID | Mm.29891 |
|  | Rn.116108 |
|  | Hs.370666 |

---

|  |  |
| --- | --- |
| Homologene ID | 1527 |

---

|  |  |
| --- | --- |
| Shape | O-vertex |

---

|  |  |
| --- | --- |
| IPI ID | IPI00289866 |
|  | IPI00314283 |
|  | IPI00311101 |
|  | IPI00194593 |

---
